# Development of Multifunctional Pullulan/Chitosan-Based Composite Films Reinforced with ZnO Nanoparticles and Propolis for Meat Packaging Applications

**DOI:** 10.3390/foods10112789

**Published:** 2021-11-12

**Authors:** Swarup Roy, Ruchir Priyadarshi, Jong-Whan Rhim

**Affiliations:** Department of Food and Nutrition, BioNanocomposite Research Center, Kyung Hee University, 26 Kyungheedae-ro, Dongdaemun-gu, Seoul 02447, Korea; swaruproy2013@gmail.com (S.R.); ruchirpriyadarshi@gmail.com (R.P.)

**Keywords:** enoki mushroom, ZnONPs, propolis, pullulan/chitosan, antibacterial, antioxidant activity, meat packaging

## Abstract

Pullulan/chitosan-based multifunctional edible composite films were fabricated by reinforcing mushroom-mediated zinc oxide nanoparticles (ZnONPs) and propolis. The ZnONPs were synthesized using enoki mushroom extract and characterized using physicochemical methods. The mushroom-mediated ZnONPs showed an irregular shape with an average size of 26.7 ± 8.9 nm. The combined incorporation of ZnONPs and propolis pointedly improved the composite film’s UV-blocking property without losing transparency. The reinforcement with ZnONPs and propolis improved the mechanical strength of the pullulan/chitosan-based film by ~25%. Additionally, the water vapor barrier property and hydrophobicity of the film were slightly increased. In addition, the pullulan/chitosan-based biocomposite film exhibited good antioxidant activity due to the propolis and excellent antibacterial activity against foodborne pathogens due to the ZnONPs. The developed edible pullulan/chitosan-based film was used for pork belly packaging, and the peroxide value and total number of aerobic microorganisms were significantly reduced in meat wrapped with the pullulan/chitosan/ZnONPs/propolis film.

## 1. Introduction

Currently, food spoilage is a growing concern of the global food industry, and food spoilage has a significant impact on food supply and demand. The main cause of food quality degradation due to the oxidation and deterioration of food is the contamination and proliferation of micro-organisms during food storage. Such food spoilage has significantly impacted the quality and safety of perishable foods, especially animal products, due to the high fat content of meat products. In recent years, the intake of processed meat products has been very high, causing a serious spoilage problem in production and consumption. According to reports, ~23% of meat produced is lost or wasted [1]. As consumers demand safe food, there is a growing interest in developing and using edible packaging to preserve meat quality to keep food safe and extend its shelf life [2]. The most important challenge in this situation is to manufacture suitable packaging materials that can extend the shelf life of food. For this purpose, active packaging with antibacterial/antioxidant functions is emerging as one of the tools to control the deterioration of food [3,4,5].

Active packaging has an advantage over the direct use of antioxidant/antibacterial ingredients in foods because this type of packaging allows for the controlled release of active ingredients from the food packaging surface [6,7,8]. In this regard, renewable and degradable bio-based polymers of natural origin are suitable candidates to replace synthetic plastics in reducing environmental pollution [9,10,11]. Currently, various bio-based polymers such as polysaccharides and proteins are being studied. Although they have disadvantages such as low strength and gas barrier properties, they attract great attention as substitutes for petroleum-based plastics [12,13,14]. Another way to address the limitations of bio-based polymer matrices is to combine biopolymers to create blend films or to create composite materials with functional nanofillers or bioactive compounds [15,16,17,18,19]. For this, the blend of pullulan and chitosan can be an ideal choice as a solid base polymer matrix. Both pullulan and chitosan are edible polysaccharide-based biopolymers known for their excellent film-making potential. Additionally, previous reports have shown that these combinations create a highly compatible film that compensates for the imperfections of each matrix [20,21,22,23,24].

Nanofillers and bioactive natural ingredients are known to enhance the functional properties (antimicrobial and antioxidant) of active packaging films. As one of the commonly used nanofillers, zinc oxide nanoparticles (ZnONPs) are generally recognized as safe (GRAS) to be used in the food sector [25,26,27,28]. Adding ZnONPs improves the antimicrobial properties and helps enhance the physical properties, like the mechanical and water vapor barrier properties, of the packaging films [29,30,31]. As a potential antioxidant filler, the bioactive natural compound propolis plays a useful role due to its flavonoids, essential oils, waxes, and pollen [32,33,34,35]. Propolis is a complex mixture mainly obtained from honey bees, and it is a well-known, naturally occurring multifunctional material used since ancient times. The combination of nanofillers (ZnONPs) and natural bioactive compound (propolis) can be a good combination to enhance the physical and functional properties of edible active packaging films. A combination of antibacterial/antioxidant functional fillers in bio-based packaging films can be particularly useful to control the spoilage of meat products.

Therefore, the current work aimed to fabricate multifunctional active packaging films based on a pullulan/chitosan blend film integrated with ZnONPs and propolis. The pullulan/chitosan-based composite film was prepared, the film properties were characterized using analytical methods, and film’s applicability to pork loin packaging was tested.

## 2. Materials and Methods

### 2.1. Materials

The pullulan was procured from Korea Bio Polymer Co. Ltd. (Bucheon, Gyeonggi-do, Korea). The zinc chloride, potassium hydroxide, and glycerol were acquired from Daejung Chemicals & Metals Co., Ltd. (Siheung, Korea). The chitosan (viscosity: 200–800 cP at 1% acetic acid, MW: 190,000–310,000 based on viscosity, degree of deacetylation: 75–85%), 2,2-diphenyl-1-picrylhydrazyl, 2,2′-azino-bis (3-ethylbenzothiazoline-6-sulfonic acid), and potassium persulfate were purchased from Sigma-Aldrich (St. Louis, MO, USA). The alcoholic extracts of propolis and enoki mushrooms were procured from a local supermarket in Seoul, Korea.

### 2.2. Preparation of Mushroom-Mediated ZnONP

For the preparation of the ZnONPs, the enoki mushroom extract (ME) was first prepared. For this, 10 g of enoki mushroom was cut into tiny pieces, mixed with 100 mL of DI water, heated at 80 °C for 10 min while being stirred at 500 rpm, and then cooled to room temperature. The undissolved solids were removed by centrifugation at 5000 rpm for 15 min. The collected extract was stored at −20 °C until further use [36,37]. A total of 6.8 g of ZnCl_2_ was added (0.1 M) to 500 mL of 10% ME, mixed well, and heated until the temperature reached 70 °C. Then, 500 mL of KOH solution (0.2 M) was slowly added to the mixture with vigorous agitation for one hour. The white principate formed was collected by filtration, and the filtrate was washed with DI water until it reached a pH of 7 and then dried at 50 °C overnight to attain powdered ZnONPs.

### 2.3. Preparation of Pullulan/Chitosan-Based Films

**Scheme 1:**
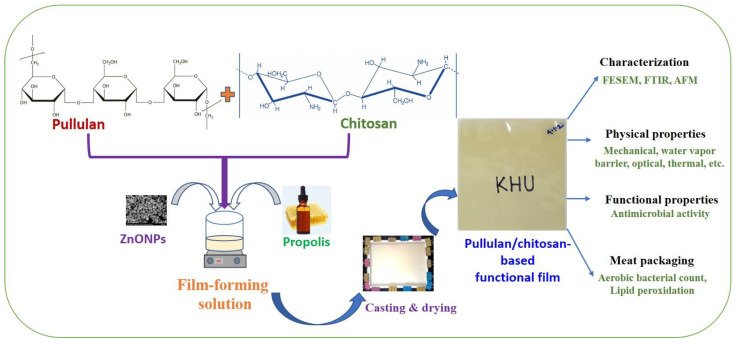
Schematic presentation for the fabrication of pullulan/chitosan-based biocomposite films.

The pullulan/chitosan-based film was prepared using a solution casting method [22], as presented in Scheme 1. For this, 1.5 g of chitosan was dissolved in 100 mL of 1% acetic acid solution with vigorous stirring. A total of 1.5 g of pullulan was dissolved in 100 mL of DI water by heating it at 70 °C with continuous agitation. Then, the chitosan and pullulan solutions were mixed, glycerol 0.9 g (30 wt% of polymer) was added, and then 0.08 g of ZnONPs and 0.2 g of propolis (2 and 5 wt% based on biopolymer, respectively) were added with vigorous stirring. The film-forming solutions were cast on Teflon film-coated glass plates. The dried films were conditioned at 25 °C and 50% RH for 72 h. For evaluation, neat films of pullulan/chitosan, pullulan/chitosan/ZnONPs, and pullulan/chitosan/propolis were also fabricated following an identical process. The prepared films were designated as PLN/CTS, PLN/CTS/ZnO, PLN/CTS/PPS, and PLN/CTS/ZnO/PPS, respectively, depending on the composite materials.

The details of the characterization methods of mushroom-mediated ZnONPs and the biocomposite films are provided in Supporting Information.

## 3. Results and Discussion

### 3.1. Characterization of ZnONPs

**Figure 1:**
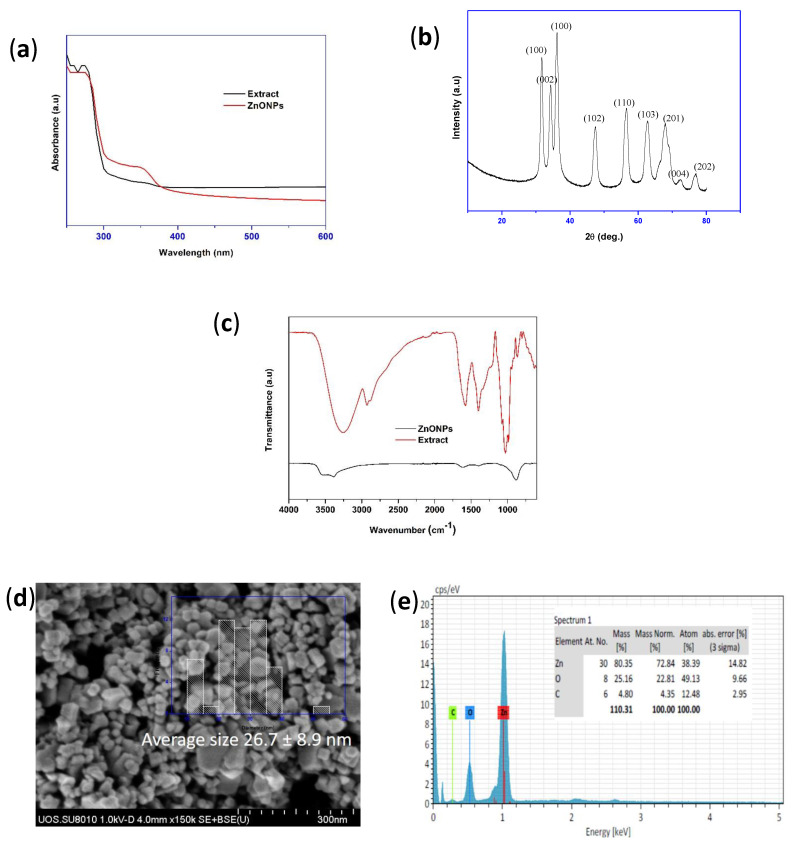
(**a**) UV-vis spectra, (**b**) XRD pattern, (**c**) FTIR spectra, and (**d**,**e**) FESEM and EDX of ZnONPs.

The appearance of white residue demonstrated the formation of ZnONPs. The formation of ME-capped ZnONPs was examined by the UV-vis spectra (Figure 1a), and the results showed a clear, typical absorption band around 350 nm due to the ZnONPs. The XRD patterns of the ZnONPs (Figure 1b) showed a distinctive pattern of diffraction peaks at (110), (002), (100), (102), (110), (103), (201), (004), and (202), representing the crystalline ZnONPs. The obtained results are well matched with the earlier reports indicating the formation of ZnONPs [30]. The chemical structure of the ZnONPs capped by ME was studied using FTIR analysis, and the FTIR results of the ZnONPs and ME are shown in Figure 1c. The wideband at 3265 cm^−1^ was owing to the primary amine and O-H stretching, while the peak detected at 2930 cm^−1^ was ascribed to the stretching vibration of the C-H groups [38]. The peak found at 1650 cm^−1^ was owing to the amide peak. Peaks at 1410 and 1035 cm^−1^ were accredited to the O-H bending and stretching vibrations, respectively [39]. The stretching vibration of primary amines and O-H is most likely associated with the bioactive components (proteins, flavonoids, phenolic acids, tannins, and carbohydrates) of the mushroom extract responsible for capping the ZnONPs [40]. The FTIR data established that the ZnONPs were efficaciously capped with several polyphenolic and biomolecules present in the mushroom extract. The observed results are also in line with the formerly available report [41].

**Figure 2:**
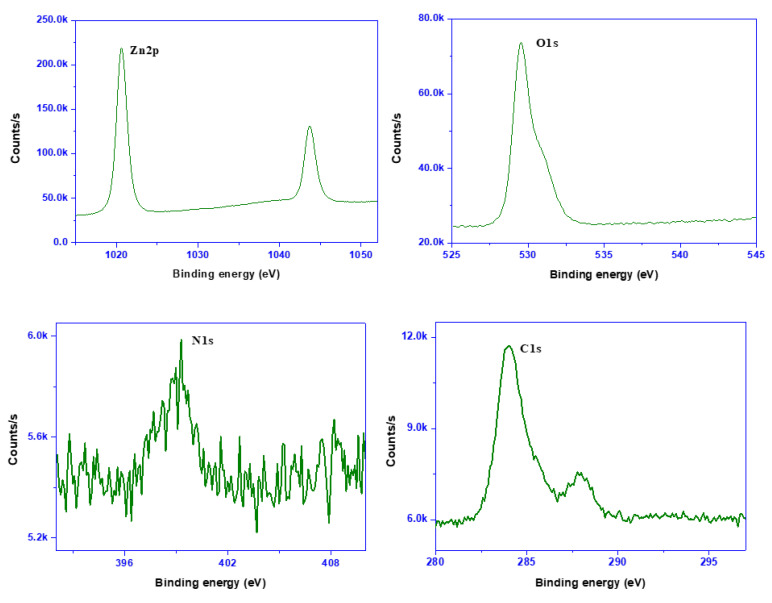
XPS spectra of ZnONPs.

For further characterization of the ZnONPs, the hydrodynamic particle size (Z_avg_) and polydispersity index (PDI) were analyzed using dynamic light scattering. The Z_avg_ and PDI were 451.3 ± 24.8 nm and 0.36 ± 0.01, respectively (Figure S1, Supporting Information), demonstrating the development of monodispersed nano-sized particles. The stability of the nanoparticles in an aqueous condition was checked by zeta potential, and the obtained data (−25.2 ± 0.5 mV, Figure S1, Supporting Information) revealed the moderate stability of the nanoparticles [42,43]. The stability of the ME-capped ZnONPs solution was very similar to the earlier reported melanin-mediated ZnONPs [30]. For the morphology of the particles, FESEM analysis was performed, and the results are presented in Figure 1d. The microscopic analysis showed irregular-shaped ZnONPs with a size of ~10–40 nm, with an average size of 26.7 ± 8.9 nm. The presence of elemental Zn and O was also further verified from the energy-dispersive X-ray spectroscopy (EDX) (Figure 1e). The elemental analysis was further confirmed by the XPS spectrum (Figure 2). The scan survey spectrum of ME-capped ZnONPs and C1s, N1s, O1s, and Zn2p are shown in Figure 2. The XPS spectra exhibited the binding energies of C1s, N1s, O1s, and Zn2p at about 284, 399, 530, and 1020 eV, respectively. Apart from the peaks of Zn and O, supplementary peaks of C and N were detected, most likely owing to the capping of ME. The results of the binding energy values for Zn2p and O1s are consistent with formerly described data [44]. Thus, the resulting characterization established the formation of ME-capped nano-sized particles.

### 3.2. Properties of the Pullulan/Chitosan-Based Composite Films

#### 3.2.1. Apparent Color and Optical Properties

**Figure 3:**
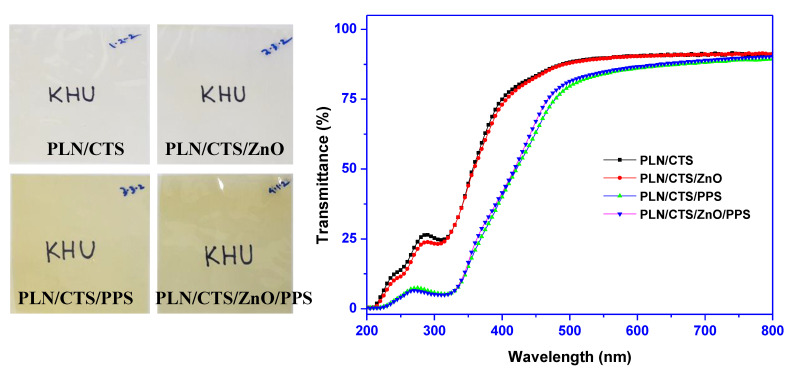
The appearance and transmittance spectra of the pullulan/chitosan-based composite films.

The appearance and light transmittance spectra of pullulan/chitosan-based binary biocomposite films are presented in Figure 3. The neat pullulan/chitosan (PLN/CTS) film and the ZnONPs-added (PLN/CTS/ZnO) film were slightly yellowish and transparent, while the film with propolis was yellowish due to the color of propolis. The light transmittance spectra of the film showed that all the films were see-through to visible light. The control films also exhibited a peak at 280 nm owing to the UV-light absorption of chitosan. The reinforcement of the film with ZnONPs slightly reduced the UV light transmittance. Nevertheless, the addition of propolis pointedly condensed the UV transmittance of the pullulan/chitosan film. The combined use of ZnONPs and propolis showed a somewhat synergistic effect in the reduction of the UV-light transmittance of the film. The film’s UV-light barrier and transparency were assessed, and the results are presented in Table 1. The neat film showed excellent transparency with more than 91% of the T_660_. The alloying of ZnONPs and propolis alone or in combination in pullulan/chitosan did not reflect any significant reduction of the transparency of the film. Nonetheless, the alloying of ZnONPs slightly reduced the UV-light transmittance (T_280_), while the mixing of propolis showed a sharp decrease in UV-light transmittance (24.7% to 7.0%) due to the polyphenolic compounds present in the propolis. The ZnONPs and propolis together somewhat synergistically decreased T_280_ to 6.4%. Hence, it can be inferred that adding the fillers makes a highly transparent film and enhances its UV-light barrier properties.

The surface color data and related color parameters are shown in Table 1. As anticipated from the UV-vis spectra, the lightness (*L*-value) of all the films was over 87%, indicating that the lightness was not considerably altered due to the reinforcement with fillers. The *a*-value was also not much transformed, although the *b*-value was amplified in the case of propolis, demonstrating increased yellowness. Accordingly, the total color difference (Δ*E*) of the films also increased significantly (*p* < 0.05) when reinforced with propolis, even if adding ZnONPs alone had shown a reduction in Δ*E*. The whiteness index (WI) showed a slight increase in the presence of ZnONPs but showed a decreasing trend in the presence of propolis, which is presumably due to the yellowness of propolis. As expected, the yellowness index (YI) of the film was significantly increased by the addition of propolis.

#### 3.2.2. Morphology

**Figure 4:**
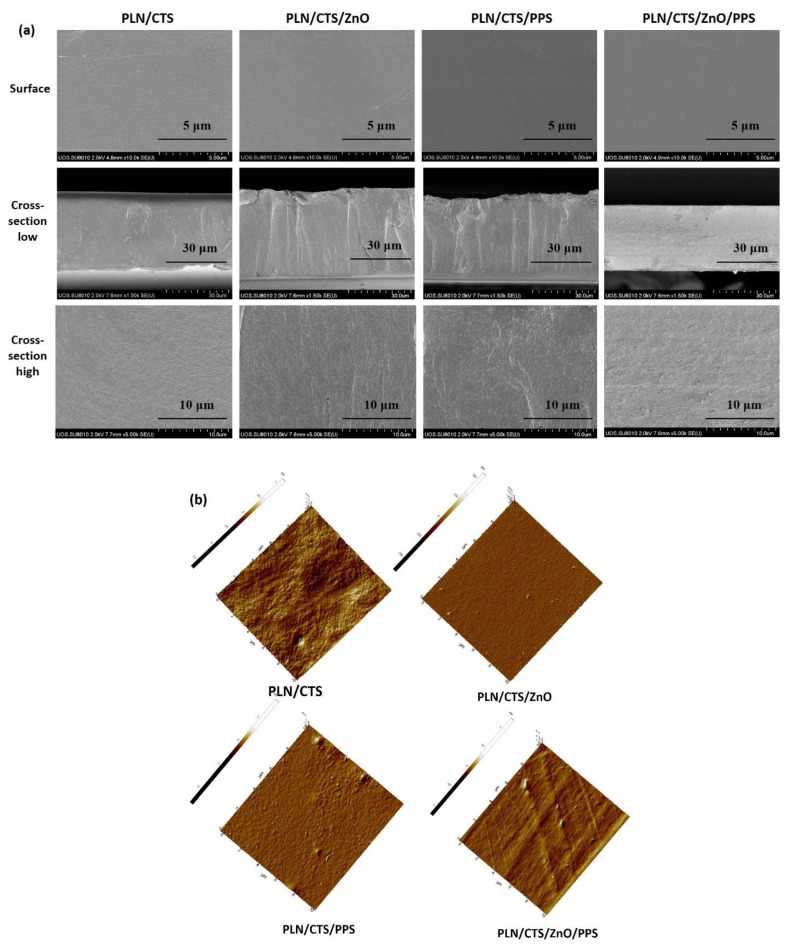
FESEM surface and cross-section micrographs (**a**) and AFM images (**b**) of pullulan/chitosan-based composite films.

The morphological structure of the film was observed using the FE-SEM (surface and cross-section) and AFM, and the data are shown in Figure 4. The SEM surface images showed all films were smooth surfaced, without any outward defects. By alloying, pullulan and chitosan produce a compatible film [22]. The reinforcement with ZnONPs or propolis did not meaningly transform the morphology of the film, indicating their appropriate miscibility in the binary polymer matrices. The cross-section morphology low and high magnification also showed a proper distribution of fillers in the polymers without significantly altering the morphology of the control films. Interestingly, incorporating ZnONPs did not induce any agglomerated particles, suggesting the proper blending of nanofillers in the polymer matrices could contribute to the good adhesion of the filler and the pullulan/chitosan matrix. For more insight into the nanofillers’ distribution, elemental mapping was carried out (Figure S2, Supporting Information), and the results showed a uniform distribution of the nanoparticles in the polymer matrices. Moreover, the morphology of the films was checked by AFM micrographs (Figure 4), and the results showed a good dispersion of the fillers analogous to the SEM data. The surface roughness of the film was also determined from the AFM micrographs, and the obtained Ra and Rq values (data not shown) indicate that the increase in roughness was observed only for ZnONPs while adding propolis did not significantly alter the roughness of the pullulan/chitosan films.

#### 3.2.3. Mechanical Properties

The mechanical properties of the pullulan/chitosan-based films were determined in terms of the tensile strength (TS), elongation at break (EB), and elastic modulus (EM), and the results are shown in Table 2. The thickness of the control blend film was 43.1 μm, which slightly decreased to 41.6 μm with the mixing of ZnONPs. This might be due to the formation of H-bonding between ZnONPs and chitosan, which may have pulled the polymer chains closer, resulting in enhanced compactness and decreased thickness [45]. However, when propolis was added to the neat blend films, the thickness increased to 47 μm. The reason for this may be the high molecular weight of polyphenols in propolis, which occupy the interchain spaces in the polymer matrix, pushing them farther apart [46,47]. Also, this phenomenon designates the absence of any chemical interaction among propolis and the polymer chains. When both ZnONPs and propolis were added, the effect of both the additives balanced each other, resulting in a thickness of 45.5 μm. Nevertheless, the variation in the thickness among different films was not statistically significant.

The TS values confirm the H-bonding interaction of the ZnONPs with the biopolymer chains. The TS of the PLN/CTS/ZnO and PLN/CTS/ZnO/PPS films were 61.5 MPa and 62.6 MPa, respectively, indicating statistically significant ~24% and ~26% increments compared to neat PLN/CTS blends. However, the PLN/CTS/PPS film showed an insignificant increase (by 4%) in the TS value. Likewise, the EM values of the the ZnONPs-incorporated films also increased to 2.6 GPa compared to 2.2 GPa for films without ZnONPs. Additionally, the EB values were decreased more for the ZnONPs-incorporated films. Nevertheless, the difference in the EM and EB values was not statistically significant, indicating minor variations in the flexibility and stiffness of the films [48].

#### 3.2.4. Water Vapor Permeability (WVP) and Water Contact Angle (WCA)

The changes in the WVP and WCA values upon incorporating ZnONPs and propolis are shown in Table 2. No statistically meaningful change in the WVP value was observed due to incorporating ZnONPs in the blend films. Although it is reported that nanoparticles, when added to the polymer matrix, generally enhance the water vapor barrier properties by occupying the vacant interchain spaces and creating a tortuous path for the water molecules to pass through [45], some reports also indicate the opposite effect due to the hydrophilicity of the ZnO nanoparticles capped with biomolecules [49]. The unperturbed WVP values may be the result of the balance between both these attributes of ZnONPs. Conversely, adding propolis enhanced the water vapor barrier properties, which is attributed to the decreased hygroscopicity of the films resulting from the increased content of hydrophobic components in the propolis [50]. The WCA values displayed a similar trend where the films with propolis displayed high hydrophobicity compared to the neat blend films. However, the ZnONPs-incorporated films displayed increased interaction with water due to their hydrophilic nature. Despite this, all the films displayed substantial increments in the WCA values. However, the films could not be classified as hydrophobic as the WCA values were not above 65°, which is the recommended WCA value for hydrophobic films [51]. Nevertheless, the PLN/CTS/PPS films exhibited a WCA value of 64.6°, making them the most hydrophobic of their counterparts.

#### 3.2.5. FTIR and Thermal Analysis

The results of the structural characterization of the films and their thermal analysis are provided in the Supplementary Information, Figures S3 and S4, respectively.

### 3.3. Antimicrobial Activity

**Figure 5:**
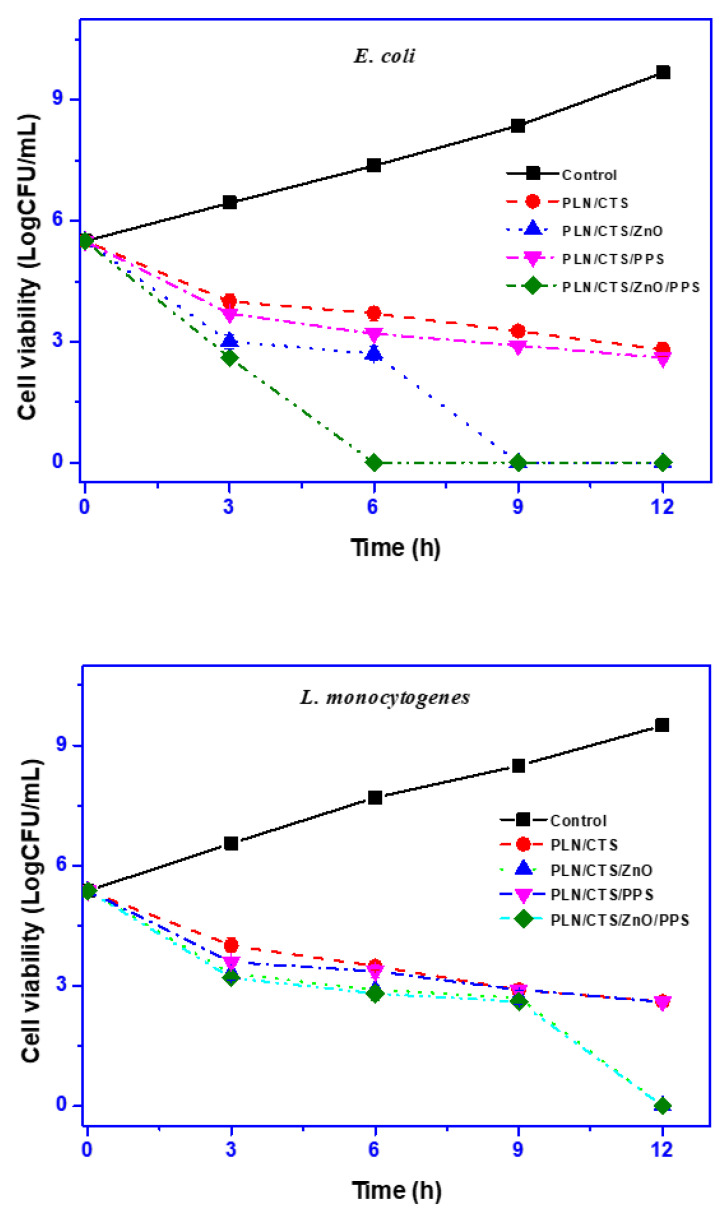
Antimicrobial activity of pullulan/chitosan-based composite films.

The antimicrobial activity of the pullulan/chitosan-based films was studied against Gram-positive *L. monocytogenes* and Gram-negative *E. coli* bacteria, and the results are presented in Figure 5. The neat PLN/CTS film displayed significant antimicrobial activity toward the tested bacteria due to chitosan’s antimicrobial action [52]. For both the bacterial strains, the growth after 12 h reached > 9 Log CFU/mL for the control group. However, for the groups exposed to the PLN/CTS films, the growth after 12 h was reduced to ~3 Log CFU/mL against both the bacteria, indicating the strong antimicrobial activity of chitosan. With the addition of propolis, no remarkable difference in the bactericidal action was observed against either bacteria, signifying no antimicrobial activity of the polyphenolic extract. Similar results were obtained previously [34]. However, as expected, the antimicrobial action of the films was boosted with the blending of ZnONPs. Against *E. coli*, a 100% bactericidal effect was observed for the PLN/CTS/ZnO film after 9 h. Interestingly, the PLN/CTS/ZnO/PPS film exerted the same effect at a 6 h interval, indicating the possible synergistic bactericidal effect of ZnONPs and propolis. However, this effect was not observed against *L. monocytogenes,* and 100% bacterial inhibition was observed after 12 h for the PLN/CTS/ZnO and PLN/CTS/ZnO/PPS films. The results also indicated a higher antimicrobial activity of ZnONPs toward Gram-negative bacteria than Gram-positive ones, which is consistent with previous reports [26,28].

### 3.4. Antioxidant Activity

**Figure 6:**
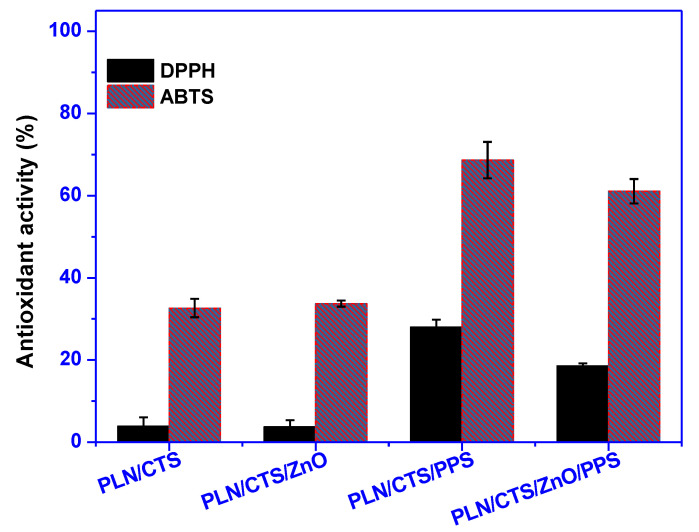
Antioxidant activity of pullulan/chitosan-based composite films.

Figure 6 shows the outcomes of the antioxidant activity of the pullulan/chitosan-based films studied against DPPH and ABTS free radicals. The PLN/CTS film showed ~5% and ~33% antioxidant activity toward DPPH and ABTS free radicals, respectively, due to the presence of chitosan. The antioxidant activity of chitosan has been reported previously and is attributed to the protonated amine group, which stabilizes the free radicals [53]. Additionally, the higher activity against ABTS compared to DPPH occurredbecause hydrophilic chitosan interacts better with ABTS, due to its aqueous solution, than with the methanolic DPPH solution [51]. With the addition of ZnONPs, no variation in the antioxidant activity was detected. However, when propolis was mixed with the pullulan/chitosan films, the antioxidant activity sharply increased to ~30% and ~70% against DPPH and ABTS, respectively. This signifies that propolis imparts a strong antioxidant property to the films due to its polyphenolic composition [34,54]. Previous studies also report the enhanced antioxidant action of bio-based polymer films upon the addition of propolis [34]. Interestingly, for the PLN/CTS/ZnO/PPS film, the antioxidant activity was slightly decreased compared to the PLN/CTS/PPS films. This may be owing to the capability of ZnONPs to bring out reactive oxygen species, including oxidative free radicals, which counter the effect of propolis. This observation was previously reported as well [28].

### 3.5. Meat Packaging Test

**Figure 7:**
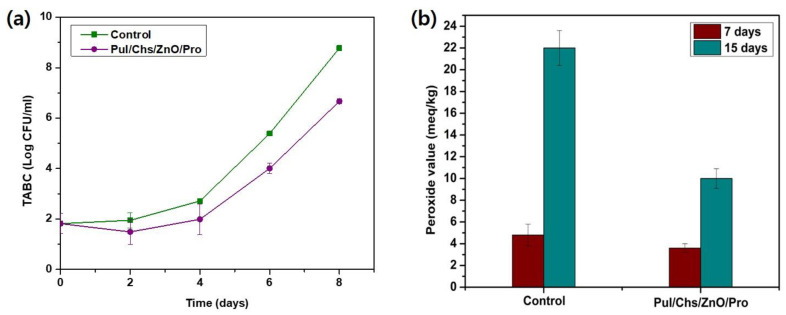
Application of pullulan/chitosan-based composite films for packaging of pork belly meat. (**a**) Total aerobic bacterial count, (**b**) peroxide values stored at 10 °C.

The commonly used preservation method for meat products is refrigeration after skin-tight packaging using PE/PP films. However, certain bacteria can grow and contaminate the packed meat even under these conditions [55]. Hence, estimating the total aerobic bacterial count (TABC) on the packed meat offers critical evidence about its hygiene and safety. The total aerobic bacterial count of the pork loin meat stored at 10 °C was estimated for 8 days, and the results are presented in Figure 7a. The initial TABC on the packed pork loin meat sample was <2 Log CFU/g. A rapid increase in the TABC was observed for the control samples while storage reached ~6 Log CFU/g after 6 days and finally ~9 Log CFU/g within 8 days. As per the International Commission on Microbiological Specifications for Foods (ICMSF) regulations, an upper acceptable microbiological limit of 7 Log CFU/g has been proposed for meat products [55,56,57]. According to this proposed limit, the packed meat in the control group was deemed consumable for up to 6 days. However, for the meat samples wrapped with the PLN/CTS/ZnO/PPS film before packaging, the TABC value remained at 6.7 Log CFU/g even after 8 days of storage, below the proposed upper acceptable microbiological limit. Additionally, this value was significantly (*p* > 0.05) lower than that obtained for the control group. Several researchers performed studies on various meat types and determined them to be consumable if the TABC value remained below 7 Log CFU/g [28,55,58,59]. Hence, it can be inferred that the PLN/CTS/ZnO/PPS film can act as an extra wrapping material that may expand the shelf life of packed pork meat, acting in the form of hurdle technology.

Besides microbial contamination, the lipid oxidation in the packed meat product is another important factor determining its quality and shelf life. The oxidation of meat lipids is inevitable during storage and transportation and renders the product rancid over time, making it unpalatable [55]. Hydroperoxides are the principal lipid peroxidation compounds that cause the development of off-flavors in food [28]. Hence, their assessment provides important evidence about the packed food quality. The peroxide values (PV) of the packed pork loin meat were determined over time, and the results are reported in Figure 7b. The initial value of lipid peroxidation in the packed meat was 0 meq/kg, which amplified over time, depending on the package type. After 7 days of storage, the control and test samples exhibited peroxide values of ~5 meq/kg and ~4 meq/kg, respectively, which were not considerably different statistically (*p* < 0.05). However, after 15 days of storing, while the PV of the meat packed in the control group increased abruptly to 22 meq/kg, the meat wrapped in the PLN/CTS/ZnO/PPS film exhibited a far lower peroxide value of ~10 meq/kg, showing around 55% decreased lipid oxidation, indicating a statistically significant (*p* > 0.05) reduction. This result was consistent with the antioxidant test results for the films (Figure 6), which indicate their oxidative free radical scavenging ability.

## 4. Conclusions

The main aim of this work was to investigate the effects of green-synthesized ZnONPs and propolis on the properties (physical and functional) of the pullulan/chitosan-based film for meat packaging applications. The ZnONPs were developed using enoki mushroom extract, and the obtained nanoparticles were stable and in a size range of 10–40 nm. Adding ZnONPs and propolis significantly enhanced the UV-light blocking and mechanical properties without significantly changing thermal stability. The alloying of fillers also slightly enhanced the water vapor barrier properties and hydrophobicity compared to the neat films. Additionally, the pullulan/chitosan-based binary composite film presented strong antibacterial activity toward *E. coli* and *L. monocytogenes* and showed excellent antioxidant activity. The PLN/CTS/ZnO/PPS film was used to wrap pork loin meat, and the TABC and PV data clearly showed that the growth of bacteria and lipid peroxidation was hindered in the packaged meat.

## Figures and Tables

**Table 1:** Surface color and transmittance of pullulan/chitosan-based bioactive composite films.

Films	*L*	*a*	*b*	Δ*E*	WI	YI	T_280_ (%)	T_660_ (%)
PLN/CTS	90.7 ± 0.3 ^c^	−2.3 ± 0.3 ^c^	13.5 ± 1.4 ^a^	9.2 ± 1.5 ^a^	86.0 ± 1.4 ^c^	21.2 ± 2.3 ^a^	24.7 ± 1.9 ^c^	91.2 ± 0.3 ^c^
PLN/CTS/ZnO	90.7 ± 0.1 ^c^	−2.1 ± 0.1 ^c^	12.2 ± 0.5 ^a^	7.9 ± 0.5 ^b^	87.2 ± 0.5 ^c^	19.2 ± 0.8 ^b^	23.1 ± 0.6 ^c^	90.6 ± 0.3 ^b^
PLN/CTS/PPS	88.1 ± 0.3 ^b^	−5.1 ± 0.2 ^a^	25.8 ± 1.1 ^c^	22.0 ± 1.1 ^d^	73.5 ± 1.1 ^a^	41.8 ± 1.8 ^d^	7.0 ± 0.4 ^b^	88.1 ± 0.6 ^a^
PLN/CTS/ZnO/PPS	87.0 ± 0.3 ^a^	−4.0 ± 0.2 ^b^	23.2 ± 1.1 ^b^	19.7 ± 1.2 ^c^	76.1 ± 1.1 ^b^	38.1 ± 2.0 ^c^	6.4 ± 0.4 ^a^	88.6 ± 0.5 ^a^

The data are denoted as a mean ± standard deviation. In the same column, any two means, followed by the same letter, are not meaningfully (*p* > 0.05) dissimilar from Duncan’s multiple range test.

**Table 2:** Mechanical properties, water vapor permeability, and water contact angle of pullulan/chitosan-based bioactive films.

Films	Thickness (μm)	TS (MPa)	EB (%)	EM (GPa)	WVP (×10^−9^ g.m/m^2^.Pa.s)	WCA (deg.)
PLN/CTS	43.1 ± 5.0 ^a^	49.7 ± 5.3 ^a^	5.9 ± 1.0 ^b^	2.2 ± 0.2 ^a^	0.77 ± 0.01 ^b^	56.5 ± 3.8 ^a^
PLN/CTS/ZnO	41.6 ± 3.9 ^a^	61.5 ± 4.3 ^c^	4.8 ± 0.9 ^a^	2.6 ± 0.3 ^b^	0.79 ± 0.03 ^b^	59.6 ± 1.6 ^ab^
PLN/CTS/PPS	47.0 ± 5.2 ^a^	51.7 ± 4.8 ^b^	5.7 ± 0.5 ^b^	2.2 ± 0.2 ^a^	0.72 ± 0.04 ^a^	64.6 ± 4.1 ^c^
PLN/CTS/ZnO/PPS	45.5 ± 4.5 ^a^	62.6 ± 4.2 ^c^	5.2 ± 0.4 ^ab^	2.6 ± 0.2 ^b^	0.69 ± 0.01 ^a^	61.3 ± 2.0 ^b^

The data are denoted as a mean ± standard deviation. In the same column, any two means, followed by the same letter, are not meaningfully (*p* > 0.05) dissimilar from Duncan’s multiple range tests.

## Data Availability

The data presented in this study are available on request from the corresponding author.

## Supplementary Materials

The following are available online at https://www.mdpi.com/article/10.3390/foods10112789/s1: Experiment, results, and discussion, Figure S1; DLS analysis (size and Zeta potential) of ZnONPs, Figure S2; Elemental mapping of PLN/CTS/ZnO/PPS film, Figure S3; FTIR spectra of pullulan/chitosan-based bioactive composite films, Figure S4; TGA and DTG thermograms of pullulan/chitosan-based bioactive composite films, References.
