# Supplementary material for: Development of Multifunctional Pullulan/Chitosan-Based Composite Films Reinforced with ZnO Nanoparticles and Propolis for Meat Packaging Applications
Source: Foods. 2021 Nov 12;10(11):2789. doi: 10.3390/foods10112789 (PMC8625050; doi:10.3390/foods10112789)
Supplement: Supplementary file 1 [file foods-10-02789-s001.zip › foods-1460235-supplementary.pdf]

foods

Supplementary Materials

Development of Multifunctional Pullulan/Chitosan-Based Composite Films Reinforced with ZnO Nanoparticles and Propolis for Meat Packaging Application

Swarup Roy †, Ruchir Priyadarshi † and Jong-Whan Rhim *

Department of Food and Nutrition, BioNanocomposite Research Center, Kyung Hee University, 26 Kyungheedae-ro, Dongdaemun-gu, Seoul 02447, Korea; swaruproy2013@gmail.com (S.R.); ruchirpriyadarshi@gmail.com (R.P.)
* Correspondence: jwrhim@khu.ac.kr
† Authors with equal contribution.

Experimental

Characterization of ZnONP

The absorption spectrum was checked using a UV-vis spectrophotometer (Mecasys Optizen POP Series UV/Vis, Seoul, Korea). FTIR spectra were noted using an FTIR spectrometer (TENSOR 37 Spectrophotometer with OPUS 6.0 software, Billerica, MA, USA). The XRD patterns were examined using a multi-purpose X-ray Diffractometer (DMAX-2500, Rigaku, Tokyo, Japan). X-ray photoelectron spectroscopy (XPS) analysis was performed using a K-alpha X-ray photoelectron spectrometer (Thermo Scientific Co. Ltd., MA, USA). The hydrodynamic particle size and zeta potential were analyzed using Zetasizer (Nano S, ZEN 1600, Malvern Instruments Ltd., UK). The morphology was examined by FESEM (FE-SEM, SU 8010, Hitachi Co., Ltd., Matsuda, Japan), the particle size was evaluated using ImageJ.

Characterization and properties of the film

Surface color and transmittance

The surface color of the films was measured using a Chroma meter (Konica Minolta, CR-400, Tokyo, Japan) with a white color plate (L = 97.75, a = -0.49, and b = 1.96) as a standard background for color measurement. The total color difference (ΔE) was calculated using the following equation:

$$\Delta E = \sqrt{(\Delta L)^2 + (\Delta a)^2 + (\Delta b)^2} \quad \text{(S1)}$$

where ΔL, Δa, and Δb are the difference between each color value of the standard color plate and film sample, respectively. The whiteness index (WI) and yellowness index (YI) of the film were calculated as follows [1,2]:

$$WI = 100- [(100-L)+ (a)^2+(b)^2]^{0.5} \quad \text{(S2)}$$

$$YI = (142.86 \times b)/L \quad \text{(S3)}$$

The UV-vis spectra of the binary composite films were recorded using a spectrophotometer (Mecasys Optizen POP Series UV-vis spectrophotometer, Seoul, Korea). The UV-barrier and transparency properties of the film were assessed by determining the percent light transmittance of the film at 280 nm (T_{280}) and 660 nm (T_{660}), respectively [3].

Surface morphology and FTIR

The film samples' surface morphology was observed using field emission scanning electron microscopy (FE-SEM, SU 8010, Hitachi Co., Ltd., Matsuda, Japan). FTIR spectra of the MNP, GSE, and film samples were noted at a wavenumber of 4000-500 cm^{-1} with

the resolution of 32 scans at 4 cm^{-1} using a TENSOR 37 Spectrophotometer with OPUS 6.0 software (Billerica, MA, USA).

The mechanical properties

First, five rectangular strips of film samples (2.54 cm × 15 cm) from each film were cut using a precision double blade cutter (model LB.02/A, metrotec, S.A., San Sebastian, Spain). The film sample's thickness was measured using a hand-held digital micrometer (Digimatic Micrometer, QuantuMike IP 65, Mitutoyo, Japan) with an accuracy of 1 μm. The film thickness was measured at five random locations of each film sample, and their average was used. The film's mechanical properties such as tensile strength (TS), elongation at break (EB), and elastic modulus (EM) were determined following the standard method of ASTM D 882-88 using an Instron Universal Testing Machine (Model 5565, Instron Engineering Corporation, Canton, MA, USA). The Instron machine was operated with an initial grip separation of 50 mm and a crosshead speed of 50 mm/min [4].

Water vapor permeability (WVP) and water contact angle (WCA)

The WVP of the binary composite films was determined gravimetrically using a WVP cup following the ASTM E96-95 standard method[5]. At first, the WVP cup was first filled with a prescribed amount of water and then covered by the films and sealed and kept in the controlled environmental chamber at 25 °C and 50% RH. After equilibration, the WVP cup's weight was measured every hour, and weight loss was calculated. The WVTR ($g/m^2.s$) was determined from the slope (linear) of the steady-state portion of weight loss of the cup versus the time curve. Then, the WVP of the films was calculated in $g.m/m^2.Pa.s$ as follows:

$$WVP = (WVTR \times L)/\Delta p \qquad (S4)$$

where L was the film's thickness (m), and Δp was the partial water vapor pressure difference (Pa) across the film.

The film's surface wettability was determined by computing the water contact angle of the film surface using a WCA analyzer (Phoneix 150, Surface Electro Optics Co., Ltd., Kunpo, Korea). The film sample was cut (3 cm × 10 cm) and then fixed on the film holder. A drop of water (~10 μL) was added to the film's surface using a microsyringe and checked the WCA.

Thermal stability

The films' thermal stability was determined using a thermogravimetric analyzer (Hi-Res TGA 2950, TA Instrument, New Castle, DE, USA). For the measurement, ~10 mg of film sample was taken in a standard aluminum pan and scanned at a heating rate of 10 °C/min in a temperature range of 30-600 °C under a nitrogen flow of 50 cm^3/min with an empty pan as a reference.

Antibacterial activity

The antibacterial activity of the binary composite films was tested against foodborne pathogenic bacteria (*L. monocytogenes* and *E. coli*) using a total viable colony count method [6]. The test microorganisms were aseptically inoculated in the TSB and BHI broth, respectively, and subsequently cultured overnight at 37 °C with agitation at 100 rpm. The inoculum was diluted appropriately, and then 100 μL of the diluted inoculum was aseptically transferred to 20 mL of TSB and BHI diluted broth (10^5 and 10^6 CFU/mL) containing 100 mg of the composite film samples and incubated at 37 °C for 12 h with agitation at 120 rpm. Samples were taken at a predetermined time interval and plated on agar plates after appropriate dilution to evaluate the viable colonies. For comparison, the same antibacterial test was performed using a film-free culture medium and a neat film as negative and positive controls, respectively.

Antioxidant activity

Antioxidant activities of the films were measured by assessing the free radical scavenging activity. 2,2-diphenyl-1-picrylhydrazyl radical (DPPH•) and 2,2′-azino-bis(3-

ethylbenzothiazoline-6-sulfonic acid) ($ABTS^{\bullet+}$) radical scavenging methods were used for the antioxidant activity assessment [6]. For DPPH analysis, a prescribed amount of methanolic solution of DPPH was freshly made, and ~50 mg of tested film sample was added in a 10 mL DPPH solution and incubated at room temperature for 30 min and measured the absorbance at 517 nm. For the ABTS assay, a prescribed amount of potassium sulfate was added to the ABTS solution, followed by overnight incubation in the dark to make the ABTS assay solution. ~50 mg of tested film samples were added to 10 mL of ABTS assay solution, incubated at room temperature for 30 min, and measured the absorbance at 734 nm. The antioxidative activity of the composite films was calculated as follows:

$$\text{Free radical scavenging activity (\%)} = \frac{A_c - A_t}{A_c} \times 100 \qquad \text{(S5)}$$

where A_c and A_t were the absorbances of DPPH and ABTS of the control and test film. All the test was performed in triplicate, and the average value was reported.

Meat packaging test

The pork loin meat purchased from a local market in Seoul, Korea, was used to test the films' effectiveness in maintaining the meat freshness. The meat was stored at -4 °C before being tested. The PLN/CTS/ZnO/PPS films were used for the packaging test as a wrapping material in the conventional meat packaging, thus acting in the form of hurdle technology. For this, the films were cut into 6 cm × 10 cm rectangular pieces, and approximately 5 g of meat sample was rolled aseptically with the films. The meat samples wrapped by the films were put individually into the polyethylene (PE) film, and skin-tight packaging was used. The packaged pork samples were stored at 10 °C for 8 days, and bacterial growth was estimated every 2 days. Total aerobic bacterial count (TABC) in pork samples was determined by a total colony count method. For this, the samples were aseptically transferred to a sterile stomacher bag with 45 mL of 0.1% peptone water and homogenized for 180 s in a stomacher (BagMixer® 400, Interscience, France). Subsequently, 1 mL aliquots of serially diluted homogenate were plated in duplicate on 3M™ Petrifilm counting plates. Plates were incubated at 37 °C for 48 h, colonies were counted, and the results were expressed in Log CFU/g. For each meat sample, unwrapped as well as wrapped with functional films, at least three experimental replicates for each test day were carried out, and the mean value was reported.

For estimating the peroxidation of lipids in pork loin samples during storage, the meat samples were prepared using the same method as the estimation of microbial growth without packaging. The meat samples rolled with the test films (PLN/CTS/ZnO/PPS) were stored at 10 °C for 15 days. The lipid peroxidation of each sample was determined at 7-day intervals following the AOCS 965.33 official method [7]. Briefly, after the meat sample was unwound from the test film and mechanically minced, the minced meat sample fat was extracted using Soxhlet extraction with n-hexane. The extracted fat was collected after removing the solvent using a rotary evaporator. Then, 1 mg of fat was taken into a 100 mL Erlenmeyer flask, a 6 mL acetic acid-chloroform mixture (3:2 v/v) was added, and 1 mL of saturated potassium iodide solution was added, mixed well, and then 6 mL of distilled water was added. For uniform mixing, the mixture was manually stirred after each addition, then 1 mL of 1% starch indicator was added to turn the mixture bluish-black. Then, the mixture was titrated against a 0.001 N sodium thiosulfate solution until the color disappeared from the solution. The peroxidation value was calculated using the following equation:

$$PV = \frac{S \times N \times 1000}{W} \qquad \text{(S6)}$$

where S is the volume of sodium thiosulfate consumed in the titration, N is the normality of sodium thiosulfate, and W is the weight of fat used. Peroxide values are expressed in milli-equivalents per kg of fat.

Statistical analysis

The film properties were measured in triplicate with individually prepared films. One-way analysis of variance (ANOVA) was performed to compare the differences among the samples, and the significance of each mean property value was determined (p < 0.05) by Duncan's multiple range test using the SPSS statistical analysis computer program for Windows (SPSS Inc., Chicago, IL, USA).

Results and discussion

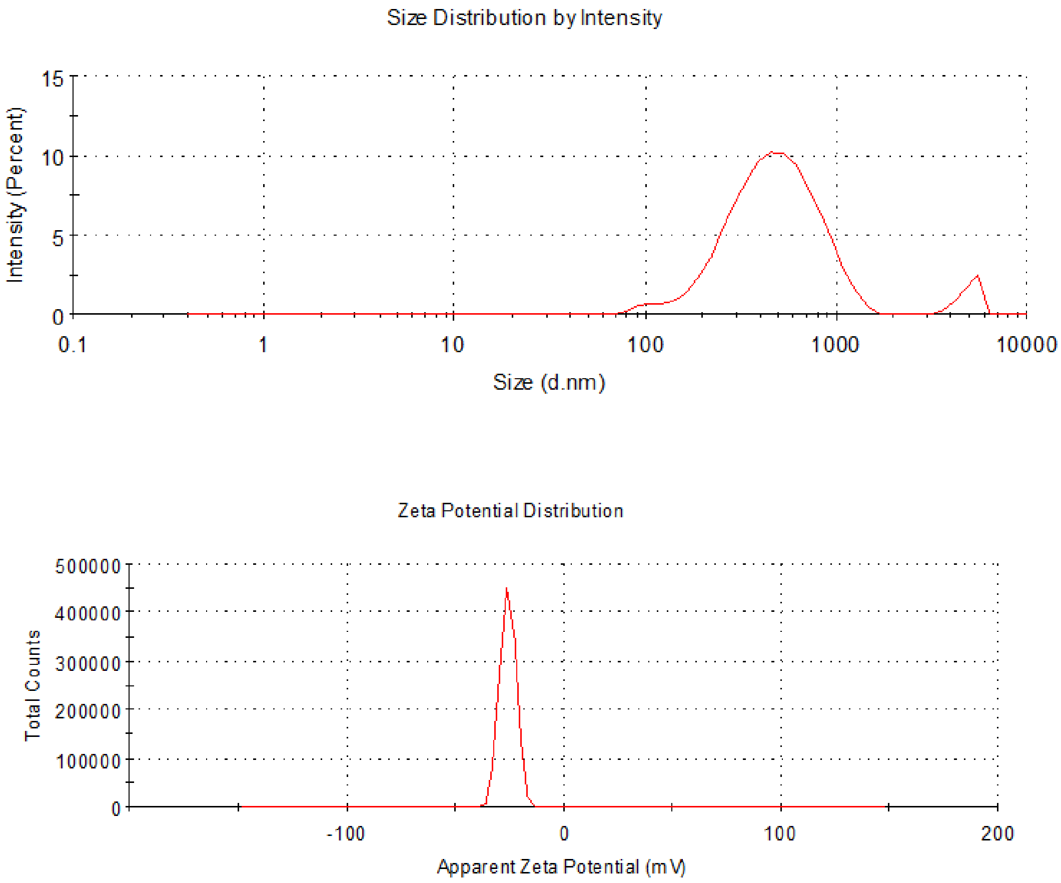

Figure S1. DLS analysis (size and Zeta potential) of ZnONP.

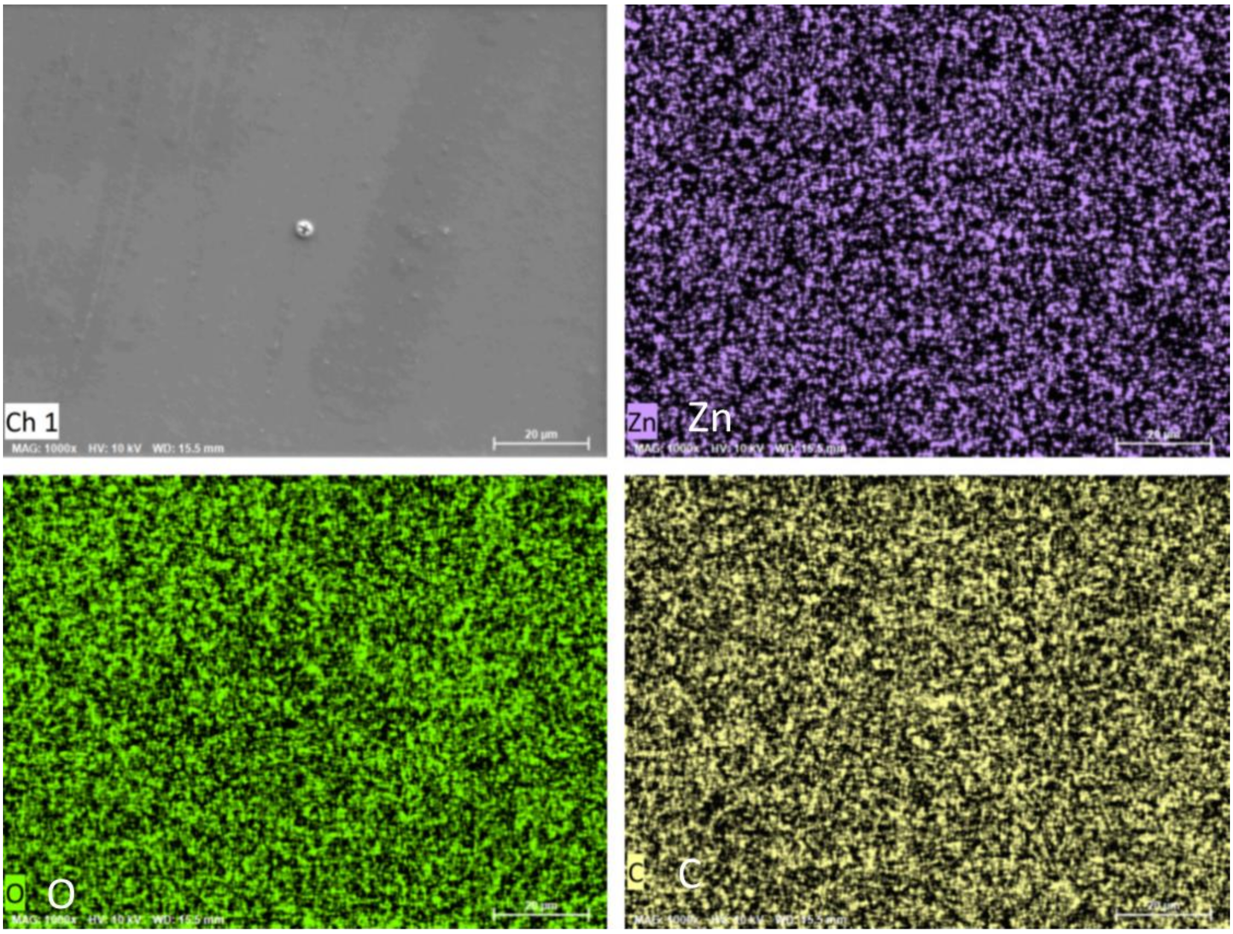

Figure S2. Elemental mapping of PLN/CTS/ZnO/PPS film.

FTIR analysis

The FTIR spectra of all the films are shown in Fig. S3. The broad peak detected for all films around 3285 cm^{-1} was ascribed to the O-H stretching vibration and intermolecular or intramolecular H-bonding [8]. The peak at 2931cm^{-1} was due to the biopolymer matrices' C-H stretching vibration (methylene and methyl group). The peak at 1643 and 1547 cm^{-1} were ascribed to -NH bending and amide-II (carbonyl stretching) and C=O stretching (amide I) of chitosan [9]. A peak detected at 1409 cm^{-1} was attributed to the O-H bending vibration of chitosan[9]. The peak observed at 1149 cm^{-1} was referred to as the glycosidic stretching vibration of pullulan [8]. The peak at 1024 cm^{-1} was due to the C-O stretching of pullulan and chitosan [8]. In the FTIR of the bioactive fillers added pullulan/chitosan composite film, identical chemical groups with minute transformation in peak position or intensity were observed, which inferred there was no noteworthy alteration in the functional groups of the composite film.

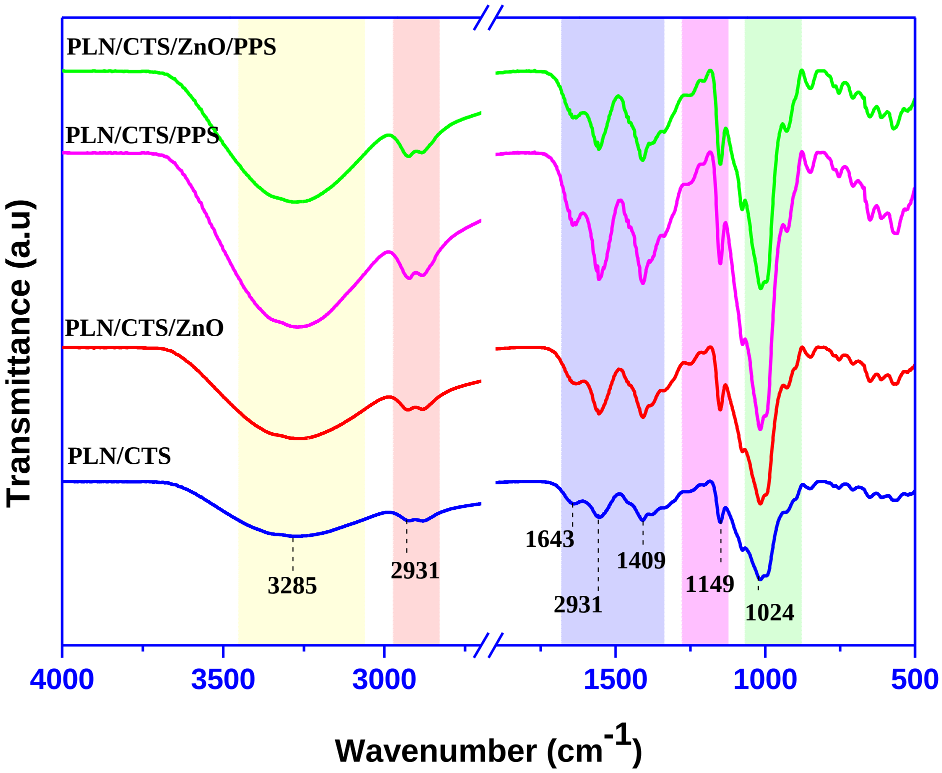

Figure S3. FTIR spectra of pullulan/chitosan-based bioactive composite films.

Thermal stability

TGA was used to check the thermal stability of pullulan/chitosan-based films, and the results are presented in Fig. S4. All the films primarily showed a two-step weight loss pattern. The initial weight loss happened at 40-125 °C owing to the loss of humidity in the film [8]. The major weight loss was detected at 130-350 °C with maximal decay at 290 °C due to the breakdown of glycerol and polymer matrices (pullulan and chitosan) [8]. Interestingly in the case of ZnONP added to film, a bifurcation of peak maximum was detected, which showed two peaks around 250 and 290 °C due to the surface functional group of ME capped ZnONP. Overall, the TGA test results showed that the alloying of fillers in polymer matrices did not meaningfully alter the thermal stability of the films. The film's final charcoal residue at 600 ℃ was 30-40%, and interestingly, the combined addition of fillers increased the char content, which might be due to the heat resistance properties of fillers (ZnONP and PE). The reasonably high residual content of the films might be ascribed to the impurities present in the polymer matrices.

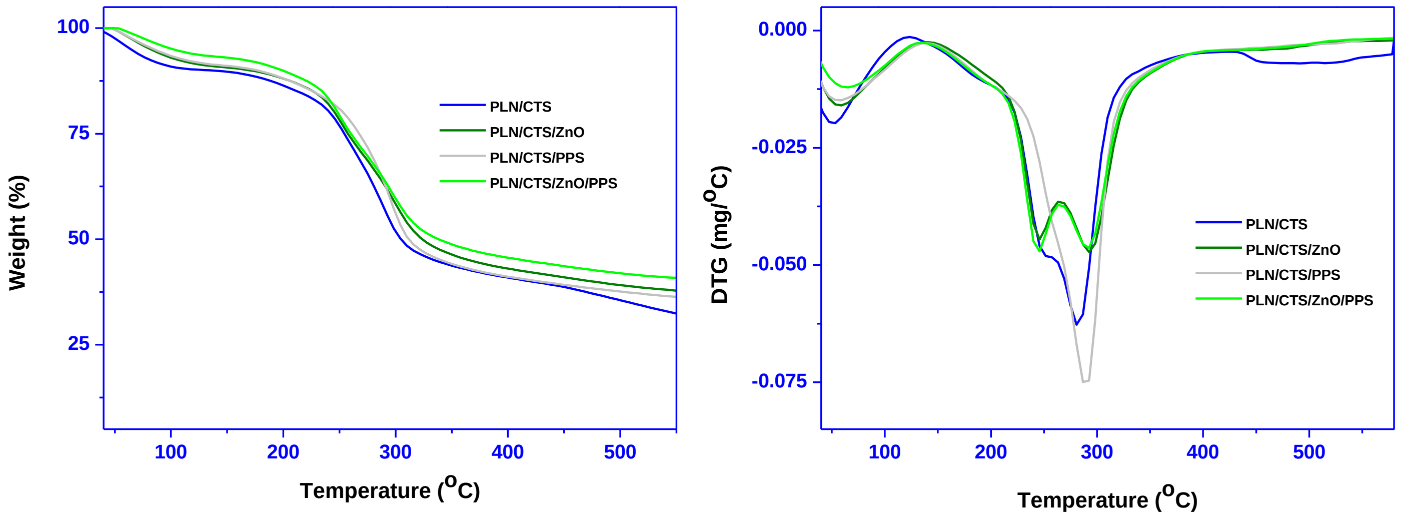

Figure S4. TGA and DTG thermograms of pullulan/chitosan-based bioactive composite films.

References

1. Roy, S.; Rhim, J.-W. Fabrication of Cellulose Nanofiber-Based Functional Color Indicator Film Incorporated with Shikonin Extracted from Lithospermum Erythrorhizon Root. Food Hydrocolloids 2021, 114, 106566, doi:10.1016/j.foodhyd.2020.106566.
2. Shao, P.; Liu, Y.; Ritzoulis, C.; Niu, B. Preparation of Zein Nanofibers with Cinnamaldehyde Encapsulated in Surfactants at Critical Micelle Concentration for Active Food Packaging. Food Packaging and Shelf Life 2019, 22, 100385, doi:10.1016/J.FPSL.2019.100385.
3. Roy, S.; Rhim, J.-W. Preparation of Gelatin/Carrageenan-Based Color-Indicator Film Integrated with Shikonin and Propolis for Smart Food Packaging Applications. ACS Applied Bio Materials 2020, 2021, 770–779, doi:10.1021/acsabm.0c01353.
4. Roy, S.; Rhim, J.-W. Carboxymethyl Cellulose-Based Antioxidant and Antimicrobial Active Packaging Film Incorporated with Curcumin and Zinc Oxide. International Journal of Biological Macromolecules 2020, 148, 666–676, doi:10.1016/j.ijbiomac.2020.01.204.
5. Gennadios, A.; Weller, C.L.; Gooding, C.H. Measurement Errors in Water Vapor Permeability of Highly Permeable, Hydrophilic Edible Films. Journal of Food Engineering 1994, 21, 395–409, doi:10.1016/0260-8774(94)90062-0.
6. Roy, S.; Kim, H.-J.; Rhim, J.-W. Synthesis of Carboxymethyl Cellulose and Agar-Based Multifunctional Films Reinforced with Cellulose Nanocrystals and Shikonin. ACS Applied Polymer Materials 2021, acsapm.0c01307, doi:10.1021/acsapm.0c01307.
7. Priyadarshi, R.; Riahi, Z.; Rhim, J.-W. Antioxidant Pectin/Pullulan Edible Coating Incorporated with Vitis Vinifera Grape Seed Extract for Extending the Shelf Life of Peanuts. Postharvest Biology and Technology 2022, 183, 111740, doi:10.1016/J.POSTHARVBIO.2021.111740.
8. Roy, S.; Rhim, J.-W. Effect of Chitosan Modified Halloysite on the Physical and Functional Properties of Pullulan/Chitosan Biofilm Integrated with Rutin. Applied Clay Science 2021, 211, 106205, doi:10.1016/J.CLAY.2021.106205.
9. Yadav, S.; Mehrotra, G.K.; Bhartiya, P.; Singh, A.; Dutta, P.K. Preparation, Physicochemical and Biological Evaluation of Quercetin Based Chitosan-Gelatin Film for Food Packaging. Carbohydrate Polymers 2020, 227, 115348, doi:10.1016/j.carbpol.2019.115348.
